# Injury mechanisms, patterns and outcomes of older polytrauma patients—An analysis of the Dutch Trauma Registry

**DOI:** 10.1371/journal.pone.0190587

**Published:** 2018-01-05

**Authors:** Rob de Vries, Inge H. F. Reininga, Oliver Pieske, Rolf Lefering, Mostafa El Moumni, Klaus Wendt

**Affiliations:** 1 Department of Trauma Surgery, University of Groningen, University Medical Center Groningen (UMCG), Groningen, The Netherlands; 2 Emergency Care Network Northern Netherlands (AZNN), Northern Netherlands Trauma Registry, Groningen, The Netherlands; 3 Department of Surgery, Evangelisches Krankenhaus Oldenburg, Oldenburg, Germany; 4 Institute for Research in Operative Medicin (IFOM), Universität Witten/Herdecke, Witten, Germany; University of Florida, UNITED STATES

## Abstract

**Background:**

Polytrauma patients nowadays tend to be older due to the growth of the elderly population and its improved mobility. The aim of this study was to compare demographics, injury patterns, injury mechanisms and outcomes between younger and older polytrauma patients.

**Methods:**

Data from polytrauma (ISS≥16) patients between 2009 and 2014 were extracted from the Dutch trauma registry (DTR). Younger (Group A: ages 18–59) and older (Group B: ages ≥60) polytrauma patients were compared. Differences in injury severity, trauma mechanism (only data for the year 2014), vital signs, injury patterns, ICU characteristics and hospital mortality were analyzed.

**Results:**

Data of 25,304 polytrauma patients were analyzed. The older patients represented 47.8% of the polytrauma population. Trauma mechanism in the older patients was more likely to be a bicycle accident (A: 17%; B: 21%) or a low-energy fall (A: 13%; B: 43%). Younger polytrauma patients were more likely to have the worst scores on the Glasgow coma scale (EMV = 3, A: 20%, B: 13%). However, serious head injuries were seen more often in the older patients (A: 53%; B: 69%). The hospital mortality was doubled for the older polytrauma patients (19.8% vs. 9.6%).

**Conclusion:**

Elderly are involved more often in polytrauma. Although injury severity did not differ between groups, the older polytrauma patients were at a higher risk of dying than their younger counterparts despite sustaining less high-energy accidents.

## Introduction

The elderly population has been increasing worldwide since 1980. In the year 2015, 17.7% of the Dutch population was older than 65 years. Increasing to 26.4% by the year 2040 [1]. However, our population is not only ageing, but the general lifestyle of the elderly is changing too. The present elderly generation shows a longer and increased mobility which contributes to a higher risk on injury. Hence, it can be expected that elderly patients presenting at trauma departments will become more frequent [2]. Moreover, it is known that the older patient has diminished physiological reserves and often inferior pre-injury functional capacities [3]. This rapidly growing ageing population will therefore impose a high burden on our healthcare system in the upcoming decades, as they are at an increased risk for receiving medical care [4–6].

Road traffic accidents are considered the most common cause of multiple traumatic injuries, also known as polytrauma. The term polytrauma is defined via an Injury Severity Score (ISS) equal to, or greater than 16. However, in the elderly minor falls (<2m, <3m) are a more common cause of polytrauma [6,7]. The described injury patterns are somewhat ambiguous. Giannoudis et al. pointed out that the younger polytrauma patient had a higher incidence of facial, neck and abdominal injuries while the elderly suffered more severe external injuries [6]. In contrast, Kocuvan et al. described the head and thorax being the most frequently injured body regions in the older polytrauma patient [7].

Multiple studies have concluded that older polytrauma patients (age ≥60 with an ISS≥16) have an increased mortality compared to the non-geriatric trauma population (28% vs. 12% [3]; 42% vs. 20% [6]; 53% vs. 27% [8]) The length of hospital stay of polytrauma survivors is prolonged for the older population, making them more vulnerable to nosocomial complications such as multi-organ failure (MOF) and sepsis, which is also associated with an increased mortality [9].

Many studies have been conducted to optimize trauma care, but its focus lay mainly on younger polytrauma patients. Large studies regarding elderly polytrauma patients are still scarce. This descriptive study has therefore analyzed data of the Dutch Trauma Registry (DTR) over a 5-year period in order to focus on the differences in injury pattern, mechanism and outcome between older (age ≥60) and younger (age 18–59) polytrauma patients.

## Methods and study population

This is a descriptive study based on data from 2009 to 2014 of the Dutch Trauma Registry (DTR). The DTR is a mandatory ongoing national trauma registry, currently gathering data from up to 98% of all Dutch hospitals [10]. All data on injured ER patients who were admitted, transferred or died within 48 hours of an accident was collected retrospectively going back to 2007.

The DTR is based on the Major Trauma Outcome Study (MTOS+) and includes patient demographics, vital signs on admission, injury mechanism, anatomical injury characteristics and outcome. All data regarding the trauma care on the ER is retrospectively collected by the DTR from the medical ER record, which is nationally standardized. Data regarding the length of stay, ICU admission and hospital mortality were collected by the DTR after admission or death.

All patients included in the DTR database from 2009 to 2014 aged 18 years and older with an ISS≥16 were included in this study. The patients were divided into two groups based on age: group A (ages 18–59) and group B (ages 60 and older). This cut-off age is based on studies regarding trauma in the older population, such as hip fractures or proximal humerus fractures [11–13]. We defined polytrauma in concordance with the worldwide accepted definition of an ISS of 16 and higher [9,14,15]. Patients who arrived dead at the ER were not included in the DTR database and therefore were not included in this study.

The local Medical Ethical Review Board reviewed the methods employed in this study and stated that this study fulfilled all the requirements for patient anonymity and was in agreement with regulations of the local University Hospital for publication of patient data (ref.nr. M17.218694). The data was de-identified prior to access and analysis.

### Injury mechanism

While the DTR is still expanding and an evolving registry, ‘injury mechanism’ has been included in the registry since 2014. The data regarding the injury mechanism therefore beholds data solely from the year 2014. The mechanism categories are: traffic accidents (motorized vehicle, bicycle, pedestrian), low-energy fall (<3 times body height) and high-energy fall (≥3 times body height), gunshot and knife injury, hit by blunt object, burn injury, and other. The “other” category beholds all mechanisms that were undefinable by the afore-mentioned categories.

### Vital signs

On admission the vital signs such as respiratory rate (RR), systolic blood pressure (SBP) and the Glasgow Coma Scale (GCS) were collected. The indication for intubation in the Netherlands is a GCS lower than 8, a compromised airway or pain requiring sedation. To give more insight into the physical state on arrival, we categorized the data according to the GCS into intubated and sedated GCS scores, and legitimate GCS scores (without intubation and sedation).

### Injury characteristics

In the DTR anatomical injury characteristics are classified by means of the Abbreviated Injury Scale (AIS). The AIS is based on systematic research of the radiology findings and injury descriptions as mentioned in the medical ER records. In order to describe the anatomical injury distribution between both groups, further defined as injury pattern, the AIS was used to classify severe injuries of specific body regions (head, face, neck, thorax, abdomen, spine, upper and lower extremity and external). An AIS ≥3 was defined as a severe injury of a specific body region. Injury Severity Score (ISS), are is also registered in the DTR [16,17].

### Outcome

Additionally, the DTR contains clinical data regarding length of stay (LOS), duration of intensive care admission and hospital mortality, these were also analyzed.

## Statistics

Statistical analyses were performed by means of IBM SPSS Statistics version 22.0 (IBM, Armonk, NY). Frequencies and proportions are presented as mean and standard deviation, or quantity and percentage. Normally distributed continuous variables’ means were tested using the independent T-test. Non-normally distributed continuous variables were tested with the Mann-Whitney U-test. Dichotomous variables’ means were tested using the Pearson Chi-square (with continuity correction) or Fischer’s Exact Test, depending on the expected count (>5: Pearson Chi-Square, <5: Fischer Exact) per cell. P-values smaller than 0.05 were defined to indicate statistical significance.

## Results

### Demographics

**Fig 1:**
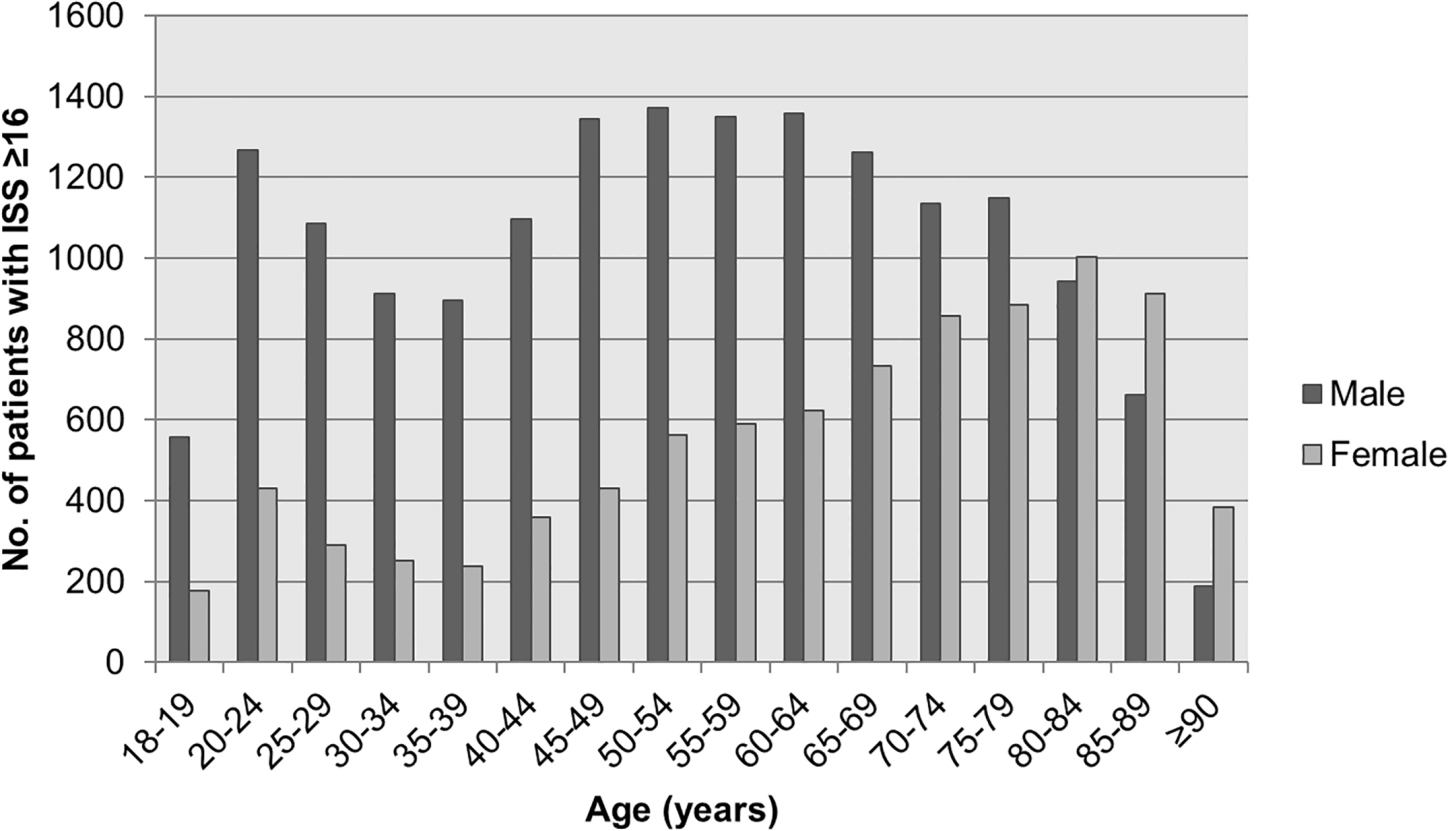
Age distribution of polytrauma patients.

Between 2009 and 2014 a total of 442,454 patients were included in the DTR database, of which 5.7% were polytrauma patients older than 18 years; 13,207 patients (52.2%) represented the younger group A (mean age: 40.1, SD: 12.8) and 12,097 (47.8%) the older group B (mean age: 75.4, SD: 9.3). The proportion of females in the younger group was 25.2%, in the older group it was 44.6%. The age distribution by gender is displayed in Fig 1.

### Injury mechanism

The mechanism of polytrauma in the older patients was more likely to be a bicycle accident (A: 17%; B: 21%) or a low-energy fall (A: 13%; B: 43%), while the younger polytrauma population was involved more often in motorized vehicle accidents (A: 29%; B: 10%) or a high-energy fall (A: 21%; B: 18%). All data on injury mechanism is presented in Table 1.

**Table 1:** Injury mechanisms (data of 2014).

	Group A age 18–59 years (n = 1,983)	Group B age ≥60 years (n = 2,235)	p-value
**Motorized vehicle**	568 (29%)	203 (10%)	<0.001
**Bicycle**	328 (17%)	474 (21%)	<0.001
**Pedestrian**	56 (3%)	66 (3%)	0.80
**Gunshot injury**	27 (1%)	5 (0%)	<0.001
**Knife accident**	54 (3%)	6 (0%)	<0.001
**Hit by blunt object**	87 (4%)	33 (1%)	<0.001
**Low-energy fall**	266 (13%)	972 (43%)	<0.001
**High-energy fall**	409 (21%)	393 (18%)	0.01
**Burn accident**	50 (3%)	19 (1%)	<0.001
**Other**	111 (6%)	64 (3%)	<0.001

### Injury severity and vital signs

The mean ISS were comparable in both groups (A: 24.4, SD: 9.8; B: 22.5, SD: 8.0). No large differences were found in vital signs such as respiratory rate and systolic blood pressure between the two groups. The younger group tended to have a worse Glasgow Coma Scale upon arrival at the hospital compared to the elderly polytrauma patients (GCS = 3; A: 20%; B: 13%). These results are presented in Table 2. The majority of these GCS = 3 scores were scored with the patient being intubated or sedated. Although the difference of intubation between younger and older trauma patients with a minimal GCS score were statistically significant, the differences were small (A: 91% vs. B: 87%). The overall intubation/sedation rate was higher among the younger polytrauma group (A: 24%; B: 16%). These results are presented in Table 3.

**Table 2:** Primary results.

	Group A age 18–59 years (n = 13,207)	Group B age ≥60 years (n = 12,097)	p-value
**Mean ISS (SD)**	24.4 (9.8)	22.5 (8.0)	<0.001
**ISS 16–24 (%)**	7,838 (59%)	7,803 (65%)	<0.001
**ISS 25–49 (%)**	4,912 (37%)	4,106 (36%)	<0.001
**ISS 50–75 (%)**	457 (3%)	188 (2%)	<0.001
**Respiratory rate (R/min)**^1^			
**0**	47 (1%)	27 (0%)	0.04
**1–5**	59 (1%)	60 (1%)	0.64
**6–9**	42 (0%)	41 (0%)	0.68
**10–29**	8,580 (95%)	7,593 (96%)	0.01
**>29**	29 (3%)	224 (3%)	0.03
**Systolic blood pressure**^2^			
**0**	63 (1%)	21 (0%)	<0.001
**1–49**	36 (0%)	13 (0%)	0.001
**50–75**	200 (2%)	159 (1%)	0.12
**76–89**	257 (2%)	195 (2%)	0.02
**>89**	11,265 (95%)	10,463 (96%)	<0.001
**Glasgow Coma Scale**^3^			
**3**	2,229 (20%)	1,267 (13%)	<0.001
**4–5**	192 (2%)	204 (2%)	0.19
**6–8**	446 (4%)	455 (5%)	0.38
**9–12**	743 (7%)	817 (8%)	0.02
**13–15**	7,476 (67%)	7,122 (72%)	0.51

^1^ 18–59: n = 9,037 (unknown: 4,170); ≥60: n = 7,945 (unknown: 4,152) (33% missing)

^2^ 18–59: n = 11,821 (unknown: 1,386); ≥60: n = 10,851 (unknown: 1,246) (10% missing)

^3^ 18–59: n = 11,116 (unknown: 2,091); ≥60: n = 9,865 (unknown: 2,232) (17% missing)

**Table 3:** Intubation and sedation rate among Glasgow Coma Scale scores.

	Group A age 18–59 years (n = 9,579)		Group B age ≥60 years (n = 8,228)		p-value
GCS	Legitimate	Tube/paralyzed	Legitimate	Tube/paralyzed	
**3**	188 (9%)	1888 (91%)	149 (13%)	1000 (87%)	<0.001
**4–5**	45 (29%)	108 (71%)	91 (58%)	65 (42%)	<0.001
**6–8**	166 (51%)	161 (49%)	215 (64%)	121 (36%)	<0.001
**9–12**	500 (86%)	79 (14%)	546 (93%)	43 (7%)	<0.001
**13–15**	6344 (98%)	100 (2%)	5943 (99%)	55 (1%)	0.001
**Total**	7243 (76%)	2336 (24%)	6944 (84%)	1284 (16%)	

18–59: n = 13,207 (unknown: 3,628); ≥60: n = 12,097 (unknown: 3,869) (30% missing)

### Injury patterns

Regarding the evaluation of each single AIS ≥3 injury in polytrauma patients, the elderly tended to have serious head injuries more often (A: 53%; B: 69%), and serious thoracic (A: 44%; B: 30%), abdominal (A: 11%; B: 3%) or severe extremity trauma (A: 28%; B: 18%) less often. Severe injuries of the face and neck were comparable in both groups. All results regarding the distribution of AIS ≥3 injuries are presented in Table 4. In order to give more insight in those polytrauma patients with a minimal GCS score a subgroup analysis of head trauma among the polytrauma patients with a minimal GCS score (GCS = 3) was performed. These results are displayed in Table 5, showing that older polytrauma patients with a minimal GCS score had more severe head injuries.

**Table 4:** Injury patterns, number of polytrauma patients with a minimum of one AIS *≥*3 per region.

	Group A age 18–59 years (n = 13,207)	Group B age ≥60 years (n = 12,097)	p-value
**Head**	7030 (53%)	8400 (69%)	<0.001
**Face**	726 (5%)	406 (3%)	<0.001
**Neck**	127 (1%)	27 (0%)	<0.001
**Thorax**	5805 (44%)	3582 (30%)	<0.001
**Abdomen**	1453 (11%)	371 (3%)	<0.001
**Spine**	1664 (13%)	1189 (10%)	<0.001
**Upper extremity**	1295 (10%)	806 (7%)	<0.001
**Lower extremity**	2330 (18%)	1338 (11%)	<0.001
**External**	364 (3%)	136 (1%)	<0.001

**Table 5:** Subgroup analysis of head trauma in polytrauma patients with a GCS score of 3.

	Group A 18–59 years (n = 2229)	Group B ≥60 years (n = 1267)	p-value
**AIS head < 3**	516 (23%)	203 (16%)	<0.001
**AIS head ≥ 3**	1713 (77%)	1064 (84%)	<0.001
**AIS head ≥ 4**	1484 (67%)	979 (77%)	<0.001

### Outcome

Table 6 shows that younger polytrauma patients were more likely to be transferred to the intensive care unit than the older patients (A: 62%; B: 45%, p<0.001). Length of stay on the ICU did not differ between the two groups. The overall hospital mortality of the older polytrauma patients was twice as high as compared to that of the younger group (A: 9%; B: 19%, p < 0.001).

**Table 6:** Outcome.

	Group A age 18–59 years (n = 13,207)	Group B age ≥60 year (n = 12,097)	p-value
**Admission duration (days, SD)**^1^	13.5 (17.7)	11.7 (13.4)	<0.001
**ICU admission (%yes)**^2^	7011 (62%)	4574 (45%)	<0.001
**ICU length of stay (days, SD)**^3^	7.8 (12.8)	7.0 (10.0)	<0.001
**Hospital mortality (%)**	1209 (9%)	2301 (19%)	<0.001

^1^ 18–59: n = 12,669 (unknown: 538); ≥60: n = 11,616 (unknown: 481) (4% missing)

^2^ ICU admission: Defined as direct transfer from ER to ICU or ≥ 1-day admission to ICU. 18–59: n = 11,353 (unknown: 1,854); ≥60: n = 10,162 (unknown: 1,935) (15% missing)

^3^ 18–59: n = 6,438 (unknown: 573); ≥60: n = 4,158 (unknown: 416) (9% missing)

## Discussion

This study pointed out that about half of all polytrauma patients is over 60 years old (47.8%). Although young polytrauma patients are involved in high-energy traumas more often, older polytrauma patients are more at risk of sustaining serious head injuries and have doubled mortality rates.

For many years trauma registries showed that polytrauma patients were mainly young males [18,19]. However, a study conducted in 2009 by Giannoudis et al. showed that the number of older polytrauma patients (≥65 years) was considerably larger than previously stated (14%) [6]. These numbers are still considerably smaller than the results from the present study. One might argue that this difference in proportion of older trauma patients might be caused by a different age cut-off point. However, the subgroup of the present study which included the polytrauma patients of 60–64 years only accounted for 8% of the total polytrauma population. The discrepancies between the results of Giannoudis et al. [6] and our study however cannot be explained only by the growth of the elderly population in the Netherlands. This considerably larger proportion of elderly could be due to the fact that this is the first large registry study describing polytrauma of the old, which is not restricted to a single medical center, as was the case for the study of Giannoudis et al. [6].

In concordance with other literature, our study also showed a shift in gender distribution between younger and older polytrauma patients. The proportion of female polytrauma patients showed an increase with increasing age (25.2% vs. 44.6%). Giannoudis et al. stated that this could be mainly clarified by two explanations: “Firstly, it is well recognized that high-risk behavior is at a peak in young males. As this behavior decreases, the proportion of victims of serious trauma who are male will likewise decrease. Secondly, higher rates of survival in aging females mean that the proportion of female patients suffering from any condition is likely to be higher in older patients” [6].

Polytrauma is often associated with high-energy traumas such as falls from considerable heights as well as motorized vehicle traumas. The present study confirms that these mechanisms account for the majority of polytrauma in the younger adult group. However, the study also showed that the mechanisms of injury in the majority of older polytrauma patients (64%) were relatively low-energy traumas, including bicycle accidents and low-energy falls. Baker et al. already concluded that low-energy falls comprised more than 50% of traumatic deaths in persons over age 65 [20]. Although this study showed no difference in injury severity between the two groups, we can conclude that it takes a smaller impact for the elderly to develop a polytrauma state compared to their younger counterparts.

This study investigated differences in injuries with AIS ≥3 of younger and older polytrauma patients. Although severe head trauma was common among younger polytrauma patients (53%), its incidence was significantly higher among older polytrauma patients (69%). A possible explanation for this difference might be the increasing atrophy of the older brain, which accelerates around age 70 and leads to a significant reduction of brain mass. This results in an increased subdural space which makes the brain more vulnerable for subdural hematomas after sustaining a closed head injury [21]. Multiple studies have concluded that the mortality caused by traumatic brain injury increases with age [22,23]. Although cause of death was not investigated in the present study, this could be one of the explanations for the doubled mortality rates in older polytrauma patients. Another explanation for the higher mortality is the increased vulnerability of older patients due to their diminished physical reserves and comorbidities. A large database analysis conducted by Frölich et al. in 2014 included 31,154 polytrauma patients and found age to be a predictive variable for developing multi-organ failure (MOF). Patients who developed MOF had a mortality risk of 34.1% compared to 7.5% among patients who did not develop MOF [9].

Younger polytrauma patients showed a higher incidence of a minimal GCS score (20% vs. 13%), suggesting that the younger polytrauma patients were more often in a worse physical state on arrival compared to the older polytrauma patients. Although the overall intubation rate for the younger group was significantly higher (24% vs. 16%), the differences in intubation on arrival within patients with a minimal GCS score were small (younger patients: 91%; older patients 87%) and it therefore does not explain the higher incidence of a minimal GCS score within the younger polytrauma patient. The differences in intubation rates could be explained by the higher incidence of thoracic injuries among the younger polytrauma patient group, which could compromise the airway or respiration.

In concordance with previous studies [3,6–8], the present study showed that older polytrauma patients had a doubled hospital mortality (19% vs. 9%) despite having a comparable injury severity score. The ISS was initially developed as a predictor of mortality among trauma patients, however the relationship might be more complex. Further research is needed to define other possible factors influencing the mortality among the older polytrauma patient.

This study has some limitations. First of all, its large database relies on registration of over 300 different hospitals within the Netherlands. Consequently data, especially physical parameters, will inevitably be partly incomplete. Secondly, data on trauma mechanisms has been included in the Dutch Trauma Registry since 2014, therefore the population size for this parameter covered only one year instead of five (n = 4,218 vs. n = 25,304). It is unlikely for this to have led to a bias in trends towards trauma mechanisms. Since this study is restricted to the DTR database, no information was available on cause of death and factors, such as comorbidities, that might have influenced the mortality rate. This study does however point out the clinical significance and growing impact these patients have on our current medical care system.

## Conclusion

Polytrauma patients are more often older than previously presumed, and make up almost half of all polytrauma patients in the Netherlands. Although injury severity did not differ between younger and older polytrauma patients, older patients showed a doubled risk of dying compared to their younger counterparts, while sustaining fewer high-energy accidents. Further research exploring the predisposing factors, complications and factors influencing mortality within the elderly polytrauma patient population is advised in order to improve trauma care.
